# Hybrid Forecast-Enabled Adaptive Crowbar Coordination for LVRT Enhancement in DFIG Wind Turbines

**DOI:** 10.3390/e28020138

**Published:** 2026-01-25

**Authors:** Xianlong Su, Hankil Kim, Changsu Kim, Mingxue Zhang, Hoekyung Jung

**Affiliations:** 1 Department of Computer Science and Engineering, Pai Chai University, 155-40 Baejae-ro, Daejeon 35345, Republic of Korea; sxl_2012@cqut.edu.cn (X.S.); hkjun@pcu.ac.kr (C.K.); zhangchenjin40@gmail.com (M.Z.); 2 School of Electrical and Electronic Engineering, Chongqing University of Technology, Chongqing 400054, China; 3 Department of Music & Sound Technology, Korea University of Media Arts, 300 Daehak-gil, Janggun-myeon, Sejong-si 30056, Republic of Korea; khg0482@pro.ac.kr

**Keywords:** DFIG, wind power forecasting, CEEMDAN, LVRT, adaptive crowbar coordination

## Abstract

This study proposes a hybrid forecast-enabled adaptive crowbar coordination strategy to enhance low-voltage ride-through (LVRT) performance of doubly fed induction generator (DFIG) wind turbines. A unified electro-mechanical model in the αβ/dq frames with dual closed-loop control for rotor- and grid-side converters is built in MATLAB/Simulink (R2018b), and LVRT constraints on current safety and DC-link energy are explicitly formulated, yielding an engineering crowbar-resistance range of 0.4–0.8 p.u. On the forecasting side, a CEEMDAN-based decomposition–modeling–reconstruction pipeline is adopted: high- and mid-frequency components are predicted by a dual-stream Informer–LSTM, while low-frequency components are modeled by XGBoost. Using six months of wind-farm data, the hybrid forecaster achieves best or tied-best MSE, RMSE, MAE, and R^2^ compared with five representative baselines. Forecasted power, ramp rate, and residual-based uncertainty are mapped to overcurrent and DC-link overvoltage risk indices, which adapt crowbar triggering, holding, and release in coordination with converter control. In a 9 MW three-phase deep-sag scenario, the strategy confines DC-link voltage within ±3% of nominal, shortens re-synchronization from ≈0.35 s to ≈0.15 s, reduces rotor-current peaks by ≈5.1%, and raises the reactive-support peak to 1.7 Mvar, thereby improving LVRT safety margins and grid-friendliness without hardware modification.

## 1. Introduction

Energy plays a foundational and strategic role in virtually all areas related to human well-being. Over the past decade, global energy demand has increased by approximately 30%, while recoverable fossil fuel reserves continue to decline. Moreover, large-scale fossil fuel consumption has resulted in severe air pollution, greenhouse gas emissions, and climate change pillar of low-carbon energy systems [1]. According to the Global Wind Report 2025 released by the Global Wind Energy Council (GWEC) [2], global newly added grid-connected wind capacity reached 117 GW in 2024 (109 GW onshore and 8 GW offshore), bringing total cumulative installations to 1136 GW across 55 countries and regions. China remains the largest contributor to this growth, with its cumulative grid-connected wind capacity reaching 521 GW by the end of 2024, marking an 18% year-on-year increase [3]. Consequently, a new type of power system with wind energy at its core is rapidly taking shape, with wind power gradually becoming the “mainstay” of the national energy mix.

As a clean and renewable resource, wind power neither consumes fossil fuels nor produces direct carbon emissions, making it widely regarded as a key solution to reduce environmental pollution and mitigate climate change [4]. However, the wind-energy conversion chain—spanning atmospheric dynamics, aerodynamic capture, electromechanical conversion, and grid-interface modulation—forms a complex, stochastic system. Influenced by factors such as climate, wind speed, atmospheric pressure, and terrain, wind power output exhibits pronounced randomness, intermittency, and volatility [5,6,7,8]. From an information-theoretic perspective, wind power time series can be seen as a high-entropy, multi-scale, nonstationary process. At the grid-integration level, such intermittency and uncertainty exacerbate operational risks for power systems and jeopardize grid security and stability [9]. In extreme cases, curtailed operations or forced shutdowns of wind farms reduce the effective utilization of wind resources [10]. Thus, balancing safety, economic efficiency, and flexibility under high wind penetration has become a central challenge for modern power systems.

From the perspective of turbine topology, commercial wind turbines are mainly categorized by drivetrain configuration into doubly fed, direct-drive, and semi-direct-drive types [11]. Direct-drive and semi-direct-drive turbines offer high power ratings and low maintenance needs, making them attractive for offshore deployment [12]. In contrast, doubly fed induction generator (DFIG) turbines remain the most common configuration for onshore wind due to their smaller size, lighter weight, cost advantages, and the absence of permanent-magnet demagnetization risk [13]. The grid integration of DFIGs relies on coordinated control of the rotor-side converter (RSC) and grid-side converter (GSC). Under deep three-phase voltage sags and other severe disturbances, crowbar protection circuits are commonly used to limit rotor overcurrent and prevent DC-link overvoltage, enabling low-voltage ride-through (LVRT). However, practical crowbar schemes are usually based on fixed current/voltage thresholds, leading to the persistent dilemma of “delayed action and inflexible release.” While such schemes ensure device survival, they may compromise re-synchronization speed, reactive support, and overall grid-friendliness during and after faults.

Meanwhile, the continuous accumulation of SCADA and meteorological data has opened the door to data science approaches for understanding and managing the inherent uncertainty of wind [14]. Wind power forecasting has become a key enabler of safe and efficient system operation, supporting production optimization, stability control, dispatching, and financial planning [15]. Wind power forecasting is typically partitioned into ultra-short-term, short-term, medium-term, and long-term tasks to meet the needs ranging from real-time control to investment assessment. As the forecasting horizon lengthens, the effects of turbulence and seasonal variability increase the uncertainty of forecasts. Methodologically, forecasting approaches span physical models, statistical models, machine learning, and deep learning. Physical and numerical weather prediction (NWP)-based methods provide valuable mesoscale information but may incur latency and error amplification in rolling operation; classical statistical models are interpretable but struggle with nonlinear, nonstationary dynamics. Recent deep-learning models and decomposition-based hybrids show strong predictive performance for complex time series. However, most of these models are optimized for point-wise loss functions and aggregate error metrics, without explicitly characterizing multi-scale uncertainty or translating forecast information into actionable control decisions for protection and LVRT.

In summary, a central scientific and engineering challenge for grids with high wind penetration is how to tightly couple high-accuracy short-term wind power forecasting with DFIG grid-integration control and protection mechanisms. In particular, there is a need to transform predictive information—such as future power levels, ramp rates, and uncertainty—into risk indicators for rotor overcurrent and DC-link overvoltage. These indicators can then be used to adapt crowbar thresholds and timing in real time. This calls for a forecast–control–protection closed loop in which the forecasting module reduces the informational entropy and complexity of the power signal, while the control/protection module exploits this information to enhance LVRT safety margins, dynamic stability, and grid-friendliness.

In this study, we use the CEEMDAN decomposition method to decompose the wind power time series and calculate the sample entropy of each intrinsic mode function (IMF) component to quantify its complexity. Sample entropy is not only used to differentiate between high- and low-frequency components of the wind power signal, but also serves as a measure of unpredictability, thus quantifying forecasting uncertainty. High sample entropy values typically correspond to unstable wind fluctuations in the short term, indicating higher forecasting uncertainty, while low sample entropy values are associated with smoother and more predictable components. Therefore, by incorporating the calculation of sample entropy, our forecasting model effectively captures the complexity of the wind power signal and quantifies forecasting uncertainty, particularly in the context of adaptive protection strategies for DFIG wind turbines, enhancing system robustness and adaptability.

Motivated by this, the present paper makes the following main contributions:

(1) Proposed Framework for Forecast–Protection–Control Closed Loop: We design a forecast-driven online crowbar adaptive-coordination scheme that maps look-ahead quantities (power, ramp rate, and residual-based uncertainty) onto two physical risk channels—overcurrent on the rotor side and overvoltage on the DC link. These channels are fused into a time-varying risk indicator that adapts crowbar triggering, holding, and release thresholds in real time. Coordinated with rotor-side current limiting and grid-side reactive-priority control, the scheme executes a practical sequence of “robust phase-lock → fast limiting → reinforced support → smooth recovery,” thereby enhancing LVRT performance and grid-friendliness without requiring additional hardware.
(2) Developed Hybrid Model for LVRT-Oriented Control: We develop a CEEMDAN → segment-wise modeling → reconstruction pipeline, in which the power series is decomposed into 11 intrinsic mode functions (IMFs). High- and mid-frequency components (IMF1–IMF6) are modeled using a dual-stream Informer–LSTM architecture (global long-sequence attention + local temporal memory), while low-frequency components (IMF7–IMF11) are fitted with XGBoost to capture smooth trends. Component forecasts are inverse-normalized and summed, and ramp rates and residual-based uncertainties are generated concurrently for direct use in risk-channel modeling. Using six months of continuous wind-farm data, the hybrid model achieves the best or tied-best MSE/RMSE/MAE/R^2^ against representative baselines, with low inference latency, meeting the requirements of online coordination.
(3) Verified Performance and Engineering Feasibility: Building upon an αβ/dq unified electro-mechanical DFIG model and dual closed-loop converter control, we explicitly formulate LVRT safety constraints and DC-link energy balance, and derive an engineering-feasible crowbar resistance range (0.4–0.8 p.u.) that balances electrical safety, thermal capacity, and grid support. In a 9 MW equivalent three-phase deep-sag simulation, the forecast-driven scheme holds the DC-link voltage within ±3% of nominal and shortens re-synchronization from ≈0.35 s to ≈0.15 s. Relative to “no/fixed-threshold crowbar” baselines, the 95% active-power recovery time is reduced, rotor-current peaks are attenuated, and reactive-support peaks are enhanced. This “simulation → hybrid forecasting → coordinated control” approach provides a reproducible engineering pathway for improving LVRT safety margins and grid adaptability in complex wind–grid systems.

This paper is structured as follows. Section 2 provides a review of related work on DFIG LVRT protection and forecasting models. In Section 3, we present the methodology used for integrating wind power forecasting with DFIG LVRT control, including the unified modeling of the DFIG system and the proposed hybrid forecasting approach. Section 4 details the experimental setup and simulation design, including the wind farm model, forecasting task, and evaluation metrics. Section 5 presents the results and analysis, demonstrating the effectiveness of the proposed method in improving LVRT performance and grid-friendliness. Finally, Section 6 concludes the paper with a summary of key findings and future research directions.

## 2. Related Work

### 2.1. DFIG LVRT and Crowbar-Based Protection

Modern wind power systems can be conceptualized as a continuous energy chain, spanning from aerodynamic capture to grid-interface modulation: incoming wind energy is converted by the rotor into shaft power, which—either directly coupled or transmitted through a gearbox—drives the generator. The resulting electrical output is then conditioned by power conversion and step-up stages to match the grid in terms of amplitude, frequency, and phase. Given long-term operation under complex meteorological and loading conditions, DFIG-based systems are exposed to a wide range of electrical and mechanical stresses, necessitating comprehensive diagnostic and protection measures.

On the diagnostic side, Bebars et al. [16] systematically reviewed methods for detecting and diagnosing internal electrical faults in DFIG wind turbines, highlighting the importance of accurately identifying stator and rotor faults under variable-speed operations. Sabzevari et al. [17] proposed a 1.9 MW DFIG control scheme that combines a voltage limiter with FO-PID- and RCO-driven RSC optimization, which enhances LVRT capability under both symmetrical (SFRT) and asymmetrical (AFRT) faults. Qi et al. [18] analyzed the impact of DFIG control parameters on power-system small-signal stability, asserting that virtual inertia and key control-parameter settings should incorporate small-signal metrics to improve modal damping and stability margins. These studies collectively reflect a shift from pure fault detection to stability-aware control parameterization.

Building on these efforts, state monitoring and fault diagnosis in DFIG systems have moved from traditional model-driven paradigms to data-driven and, increasingly, to physics–data fusion via digital twins. Equivalent-circuit and multiphysics models support explainable health assessments, while SCADA data, together with high-rate vibration, electrical, and meteorological measurements, enable anomaly detection, degradation identification, and life estimation. The digital twin approach emphasizes not only detecting anomalies but also transforming them into executable control actions, thus closing the loop from data governance and real-time inference to control execution.

Simultaneously, Karad and Thakur [19] reviewed the evolution of control strategies for DFIG systems, documenting a shift from classical PI/PID regulators to robust control methods (e.g., sliding-mode control and model predictive control), soft-computing techniques, and fractional-order controllers. They also underscored the importance of hardware-in-the-loop (HIL) and rapid-control-prototyping (RCP) platforms for real-time validation under grid conditions, such as LVRT/fault ride-through (FRT) and power-quality constraints, thus translating diagnostic and modeling advances into practical control laws and execution channels.

At the mechanistic level, DFIGs are highly sensitive to grid faults due to their topology: the stator is rigidly connected to the grid, while the rotor is interfaced through a back-to-back converter. During voltage sags, the free component of the stator flux excites pronounced transients, leading to rotor-side overvoltage/overcurrent, while a power mismatch between the RSC and GSC elevates the DC-link voltage. If transient constraints are insufficient, converter protections may be triggered, and control could be lost. Concurrently, deviations in electromagnetic torque and speed can amplify electromechanical oscillations, heightening the risk of disconnection. To address these issues, two technical pathways are commonly pursued: (i) refined control strategies and (ii) fail-safe add-on circuitry.

Within the first category, Chen et al. [20] analyzed the transient stability of the DC-link voltage during LVRT for DFIG-based turbines and proposed an improved control scheme that combines additional damping with slip-power feedforward, effectively mitigating DC-link overvoltage under fault conditions. Within the second category, Shuaibu et al. [21] reviewed FRT protection techniques for grid-connected DFIGs, systematically comparing crowbar, STATCOM/DSTATCOM, DVR, and SFCL/FCL solutions. They advocated for integrating fault-current-limiting capability into a multifunctional dynamic voltage restorer (DVR-FCL) to provide both voltage compensation and equipment protection during deep sag and swell conditions. More broadly, Tripathi et al. [22] and others have emphasized that crowbar-based hardware protection remains a widely used and economically attractive solution in practice.

Recent LVRT research has also moved beyond “single-turbine survival” towards “system-level support.” Din et al. [23] argued that under weak-grid conditions and diverse grid codes, LVRT and HVRT should be advanced in parallel, with a balanced trade-off achieved through coordinated hardware–software solutions. Moving forward, achieving robustness and reproducibility under weak grids and complex fault sequences requires reachability and controllability across the entire fault process: integrating feedforward and feedback, positive/negative-sequence decoupling, and energy management within device capability limits. It also involves building layered closed loops spanning machine-side, grid-side, and plant-level controls, while strengthening real-time, interpretable tuning of protection parameters and timing in engineering practice. However, most existing schemes still rely on fixed crowbar thresholds and fixed holding times, without exploiting real-time predictive information regarding upcoming wind power dynamics or explicitly modeling the uncertainty and informational content in the driving signals.

### 2.2. Wind Power Forecasting and Hybrid Deep-Learning Models

With the continuous accumulation of operational data, innovations in data science have provided new tools for understanding wind stochasticity and designing countermeasures, offering the potential for breakthrough advances in the wind industry. Enhancing the commercial competitiveness of wind power requires a thorough understanding of generation reliability, which is influenced by both the uncertainty of wind resources and turbine productivity. The latter depends not only on the turbine’s ability to convert wind into electrical energy during operation but also on its availability and reliability. All of these aspects call for data-science solutions to support decision-making in wind power forecasting [24].

Physical methods use geographic context and meteorological variables (e.g., temperature, humidity, and pressure) to build wind-power forecasting models [25]. These methods include numerical weather prediction (NWP) approaches [26] and spatial-correlation methods [27]. NWP is suitable for medium- and long-term forecasting scenarios and is widely used in practice; however, its reliance on high computational costs and long processing chains can introduce latency and error amplification in fast rolling forecasts and under extreme weather conditions [28]. Spatial-correlation methods exploit inter-site correlations but require extensive historical data to capture complex spatiotemporal patterns.

Statistical methods, such as ARIMA, SARIMA, and linear regression, are better suited for short-term forecasting and model temporal dependence based on historical observations. These methods are interpretable but often struggle with the nonlinear and nonstationary dynamics of wind power data [29,30]. In cases with insufficient prior data or training information, some machine learning methods may even underperform relative to simple statistical baselines [31]. With advances in artificial intelligence, research has increasingly shifted toward deep learning techniques that automatically extract complex spatiotemporal patterns from raw data, showing strong suitability and transferability for high-dimensional time series such as wind speed and wind power [32].

Within deep learning-based forecasting, hybrid and decomposition-based frameworks have attracted increasing attention. Zhang et al. [33] used NWP data combined with measured data as inputs, clustered via t-SNE and K-means, and trained an attention-based Seq2Seq model for each cluster, achieving performance superior to Random Forest and other baselines. Chen et al. [34] integrated seasonal decomposition and wavelet transform into an LSTM-based framework for long-term wind power forecasting (dual-channel LSTM + stochastic optimization); they demonstrated that this method outperformed baseline models such as GRNN and provided more reliable forecasts for long-term generation planning and operational maintenance/trading decisions. Cui et al. [35] proposed an ultra-short-term wind power point-and-interval forecasting model in which an improved sparrow search algorithm (ISSA) optimizes variational mode decomposition (VMD), and an ISSA-BiGRU-Attention model is used for point forecasting, achieving better overall accuracy than comparative methods.

Meka et al. [36] developed a short-term, multi-step forecasting framework for total wind-farm power based on temporal convolutional networks (TCN), integrating meteorological tower observations and optimizing hyperparameters via an orthogonal array tuning method. This framework outperformed LSTM, CNN+LSTM, and multivariate linear regression baselines. Zhu et al. [37] proposed a short-term hybrid wind power forecasting model that used CEEMDAN for decomposition, followed by SVR optimized via GridSearchCV to produce initial predictions, with TCN-based residual correction using correlated SCADA features. This model outperformed multiple baselines in terms of accuracy. Hu et al. [38] proposed a CEEMDAN–LSTM–TCN ultra-short-term wind power forecasting model: CEEMDAN first performs denoising and stabilization, followed by LSTM/TCN submodels to target different time scales, yielding performance gains over standalone LSTM and TCN. Yang et al. [39] combined CEEMDAN, coarse-to-fine (FTC) reconstruction, and parallel CNN–LSTM for short-term wind power prediction, while Tian et al. [40] proposed an IVMD-FE-Ad-Informer hybrid model: VMD optimized by MIC adaptively decomposes the wind power series, fuzzy entropy (FE) reconstructs subseries, and an Informer with adaptive robust loss performs predictions, achieving significantly better accuracy than multiple baselines on datasets from China and Spain.

Collectively, these works demonstrate that multi-resolution decomposition (e.g., EMD/CEEMDAN/VMD) combined with deep or attention-based sequence models can effectively mitigate nonstationarity, reduce mode mixing, and enhance predictive performance. Entropy-based metrics, such as fuzzy entropy, have also been used to guide reconstruction implicitly, leveraging information-theoretic notions of complexity. However, most existing hybrid forecasters are optimized primarily for improving conventional error metrics (MSE, RMSE, MAE, R^2^) on standalone prediction tasks. They rarely quantify how decomposed components differ in complexity and information content, nor do they explicitly integrate forecasted power levels, ramp rates, and uncertainties into the design of protection schemes, such as crowbar-based LVRT.

In particular, the problem of mapping forecasting outputs into physically meaningful risk channels, such as rotor overcurrent and DC-link overvoltage, and closing the loop to adaptive crowbar coordination, remains largely unexplored. This gap motivates the development of the forecast-driven LVRT and crowbar strategy proposed in this paper.

### 2.3. Summary of Research Gaps and Problem Formulation

In summary, several gaps remain in the current state of research, specifically the lack of unified frameworks that: (i) explicitly model the dynamics of the DFIG–converter–crowbar system and LVRT constraints within a comprehensive electro-mechanical framework; (ii) reduce the entropy and multi-scale complexity of wind power signals through decomposition, while assigning model capacity according to the complexity of individual components; (iii) quantify forecast-driven risk indicators for rotor overcurrent and DC-link overvoltage; and (iv) close the loop by adaptively coordinating crowbar actions in real-time based on these risk indicators.

This paper seeks to address the following key challenge: Given a fixed DFIG and crowbar hardware configuration, how can short-term wind power forecasts—along with their ramp rates and uncertainties—be transformed into normalized risk indices for overcurrent and overvoltage? Additionally, how can these indices be used to adapt crowbar thresholds and timing in real time, thus improving LVRT safety margins and grid-friendliness?

To solve this challenge, we propose a forecast–protection–control closed loop, integrating unified DFIG modeling, CEEMDAN-based hybrid forecasting, and forecast-driven adaptive crowbar coordination.

## 3. Methodology

### 3.1. Unified DFIG Modeling and LVRT-Oriented Control

To analyze the interaction between wind turbines and the grid under voltage disturbances, we establish a unified electro–mechanical model of the DFIG wind turbine in the stationary αβ and synchronous dq frames. The model couples stator and rotor electromagnetic dynamics, DC-link energy balance, and the mechanical dynamics of the drive train.

#### 3.1.1. Electrical Model and DC-Link Dynamics

Under stator-flux orientation, the dq-frame voltage equations of the DFIG can be written in compact form as:

(1) $$\left\{\begin{array}{c} v_{sd}=R_{s}i_{sd}+\frac{d\psi_{sd}}{dt}-\omega_{s}\psi_{sq}\\ v_{sq}=R_{s}i_{sq}+\frac{d\psi_{sq}}{dt}-\omega_{s}\psi_{sd}\\ v_{rd}=R_{r}i_{rd}+\frac{d\psi_{rd}}{dt}-\omega_{r}\psi_{rq}\\ v_{rq}=R_{r}i_{rq}+\frac{d\psi_{rq}}{dt}-\omega_{r}\psi_{rd} \end{array}\right.$$

where $v_{sd}$, $v_{sq\;}$, and $\;v_{rd}$, $v_{sq}$ are stator and rotor voltages, $i_{sd}$, $i_{sq\;}$, $i_{rd}$, $i_{rq}$ are corresponding currents, $\psi_{sd}$, $\psi_{sq}$, $\psi_{rd}$, $\psi_{rq}$ are flux linkages, $R_{s}$, $R_{r}\;$ are stator and rotor resistances, and $\omega_{s}$, $\omega_{r}$ are stator and rotor angular frequencies, respectively. Flux linkages are expressed via stator/rotor inductances and mutual inductance in the standard way.

The GSC is a three-phase two-level voltage-source converter that regulates the DC-link voltage and controls the exchange of active and reactive power with the grid. In the synchronous dq frame aligned with the grid voltage, its inner-loop current controller tracks the d/q current references, while the outer loop regulates the DC-link voltage. The DC-link capacitor dynamics are governed by the power balance between the RSC and GSC:

(2) $$C_{dc}\frac{{dV}_{dc}}{dt}=\frac{\left(P_{RSC}-P_{GSC}\right)}{V_{dc}}$$

where $C_{dc}$ is the DC-link capacitance, $V_{dc}\;$ is the DC-link voltage, and $P_{RSC}$, $P_{GSC}$ denote the instantaneous powers on the RSC and GSC sides, respectively. Equation (2) plays a key role in DC-link overvoltage risk analysis and crowbar design.

**Figure 1:**
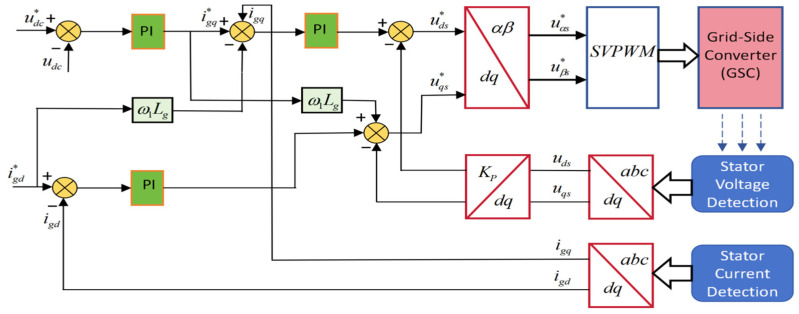
The control strategy by the side of the grid.

Figure 1 depicts the control structure of the grid-side converter, with a DC voltage outer loop and a d/q current inner loop.

#### 3.1.2. Rotor-Side Vector Control and Crowbar Interface

On the machine side, the stator is directly connected to the grid, while the rotor is interfaced through a back-to-back converter. Under stator-flux orientation, the stator’s active and reactive powers are approximately proportional to the q- and d-axis stator currents, respectively. As such, the rotor-side converter (RSC) regulates the rotor voltages in the dq frame to track the desired stator currents, and thus the active and reactive powers. This enables maximum power point tracking (MPPT) and independent active/reactive power control during normal operation.

**Figure 2:**
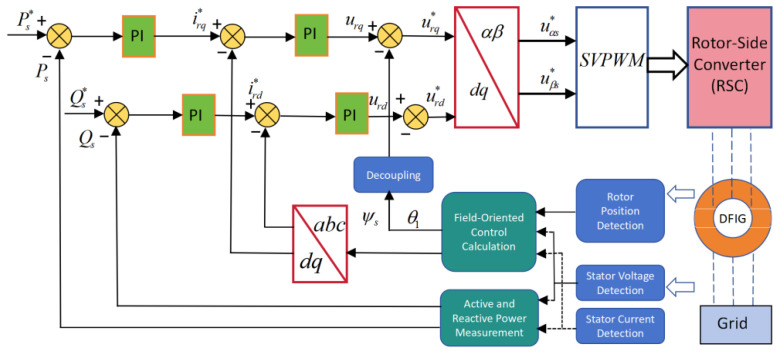
The control strategy by the side of DFIG.

To protect the RSC and DC-link under severe grid faults, a crowbar circuit is inserted into the rotor circuit. When the rotor current or DC-link voltage exceeds predefined thresholds, the crowbar is triggered, the RSC is blocked, and the rotor windings are effectively short-circuited through the crowbar resistance. The selection of the crowbar resistance must balance three objectives: (i) effective suppression of rotor overcurrent, (ii) avoidance of DC-link overvoltage, and (iii) preservation of sufficient reactive support capability. Parameter scans using the unified model indicate that a feasible engineering range of 0.4–0.8 p.u. for the rotor-side base resistance offers a good compromise; this range is used in subsequent simulations. Figure 2 illustrates the overall rotor-side control system.

### 3.2. CEEMDAN-Based Hybrid Wind Power Forecasting

To cope with the high entropy, nonstationarity, and multi-scale characteristics of wind power time series, we adopt a decomposition–prediction–reconstruction framework based on complete ensemble empirical mode decomposition with adaptive noise (CEEMDAN), combined with a dual-stream Informer–LSTM and a low-frequency XGBoost model.

#### 3.2.1. CEEMDAN Decomposition and Entropy Analysis

Let $x\left(t\right)$ denote the original wind power time series. CEEMDAN decomposes $x\left(t\right)$ into K intrinsic mode functions $\left(IMFs\right)$ and a residual term:

(3) $$x\left(t\right)=\sum_{k=1}^{K}{IMF}_{k}\left(t\right)+r\left(t\right)$$

where high-index $\left(IMFs\right)$ capture progressively lower frequency components, and $r\left(t\right)$ represents the long-term trend. In this work, CEEMDAN yields 11 $\left(IMFs\right)$ plus a residual for the six-month dataset.

To quantify the complexity of each IMF, we compute the sample entropy (SampEn). For a time series ${\left\{u\left(i\right)\right\}}_{i=1}^{N}$, SampEn is defined as

(4) $$SampEn\left(m,r,N\right)=-ln\frac{A\left(m,r\right)}{B\left(m,r\right)}\;$$

where m is the embedding dimension, r is the tolerance, and $A\left(m,r\right)$, $B\left(m,r\right)\;$ denote the counts of template matches of length m+1 and m, respectively, within tolerance r.

**Figure 3:**
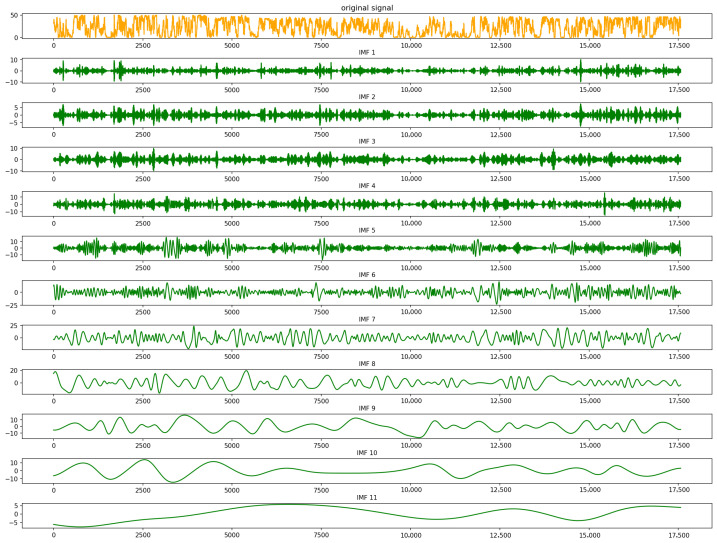
CEEMDAN decomposition.

Figure 3 shows the decomposition of the six-month wind power series into IMF1–IMF11 and residual. Table 1 summarizes the sample entropy and complexity ranking of all IMFs. The results indicate that IMF1–IMF3 have the highest entropies and correspond to high-frequency noise and fast fluctuations; IMF4–IMF6 exhibit medium entropies and capture sub-hour to multi-hour variations; IMF7–IMF11 have lower entropies and mainly reflect intra-day to seasonal trends.

This “fast–slow–noise–trend” structure naturally motivates a differentiated modeling strategy: high- and mid-frequency IMFs are assigned to high-capacity models for complex temporal patterns, while low-frequency IMFs are modeled by lightweight regression to capture smooth trends.

#### 3.2.2. Hybrid Forecasting Architecture

**Figure 4:**
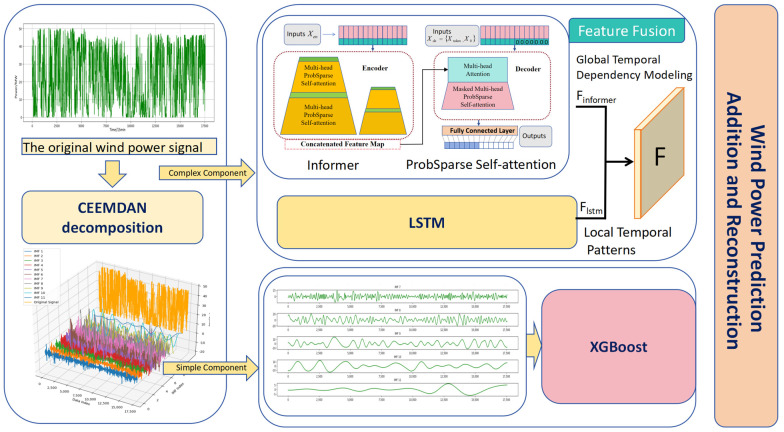
Wind Power Prediction Model Architecture.

Building on the above decomposition, we propose a dual-channel hybrid forecasting model. The overall architecture is shown in Figure 4.

The high-frequency (HF) channel processes IMF1–IMF6 using a dual-stream Informer–LSTM architecture: An Informer branch models long-range temporal dependencies with efficient global attention (as in the original Informer-based wind forecasting model). An LSTM branch focuses on local temporal memory and short-term dynamics.

The low-frequency (LF) channel processes IMF7–IMF11 using XGBoost regression trees to represent low-frequency trends and slowly varying components.

Explanation of Component Allocation: High-frequency components (IMF1–IMF6) are assigned to the Informer-LSTM model because they involve fast fluctuations and require capturing both short-term dynamics and long-term dependencies. Low-frequency components (IMF7–IMF11) are allocated to the XGBoost model, which excels at modeling smooth trends and slow variations. This allocation leverages the strengths of each model, resulting in improved forecasting performance.

Let $z_{t}$ denote the input feature vector at time t formed from CEEMDAN components and auxiliary variables. The LSTM hidden state $h_{t}$ is updated according to a simplified form

(5) $$h_{t}=LSTM\left(z_{t},h_{t-1};\theta_{LSTM}\right)$$

where $\theta_{LSTM}\;$ collects the LSTM parameters. The Informer branch encodes and decodes the input sequence using probabilistic sparse self-attention; its output at the forecast horizon is denoted by $y_{t}^{Inf}$. The two HF branches are fused by a linear combination:

(6) $${\hat{y}}_{t}^{HF}=\alpha y_{t}^{Inf}+\left(1-\alpha \right)y_{t}^{LSTM}$$

with a trainable weight α ∈ [0, 1]. For brevity, we do not assign a separate equation number to this fusion step.

For each LF IMF, XGBoost constructs an additive model of regression trees:

(7) $${\hat{y}}_{t}^{HF}=\sum_{j=1}^{J}f_{j}\left(z_{t}\right),\;f_{j}\in F$$

where each $f_{j}$ is a regression tree in the function space F. Training minimizes a regularized objective that balances data fit and model complexity.

Finally, IMF-level forecasts are inverse-normalized and summed to obtain the overall power forecast ${\hat{y}}_{t}$:

(8) $${\hat{y}}_{t}=\sum_{k=1}^{K}f_{j}{\left(IMF\right)}_{K}\left(t\right)+\hat{r}\left(t\right)$$

**Figure 5:**
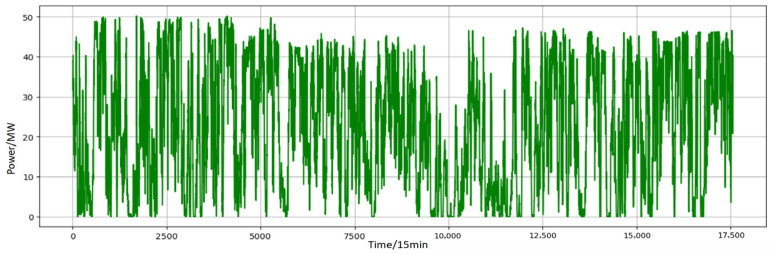
Original wind power sequence before data preprocessing.

**Figure 6:**
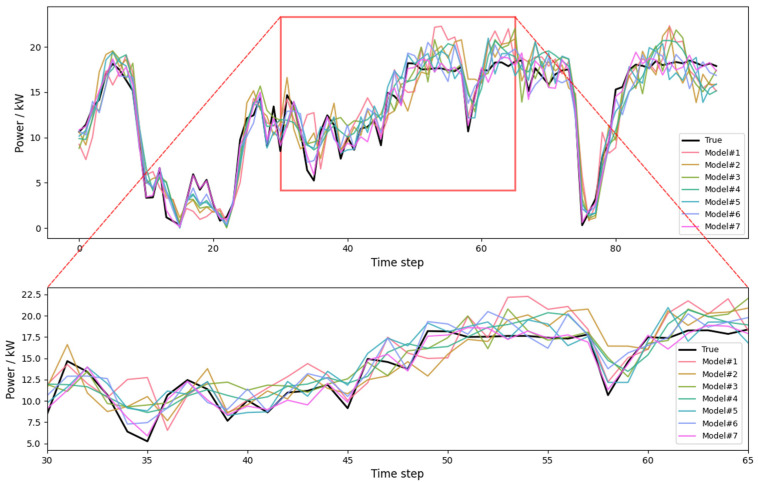
Comparison of Wind Power Prediction Results from Multiple Models. The upper panel shows the overall prediction results, while the lower panel presents a zoomed-in view of the highlighted time window. Model #1–#7 denote different forecasting models used in this study.

**Figure 7:**
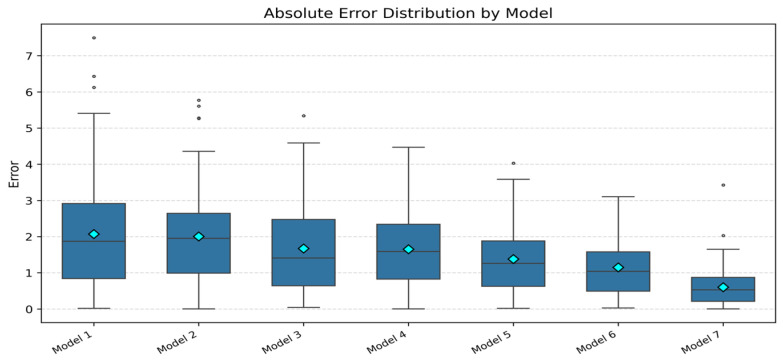
Distribution of Absolute Prediction Errors by Model for Wind Power.

Figure 5 shows the raw wind power time series over the six-month period, while Figure 6 and Figure 7 present representative forecast comparisons and error distributions for different models.

### 3.3. Forecast-to-Risk Mapping and Adaptive Crowbar Strategy

The key innovation of this work is to map short-term wind-power forecasts into rotor-overcurrent and DC-link-overvoltage risk indicators, and to use these indicators to adapt crowbar thresholds and timing online.

#### 3.3.1. Forecast-Driven Risk Channels

Let $X_{t}$ denote the historical SCADA feature vector at time t (including wind speed, direction, power, and meteorological variables). The hybrid forecaster outputs a point prediction of active power over a look-ahead window [t, t + Δ]; the predicted power at horizon Δ is denoted by

(9) $$\hat{P}\left(t+\Delta \right)=f_{hyb}\left(X_{t};\theta \right)$$

where $f_{hyb}$ represents the CEEMDAN–Informer–LSTM–XGBoost model and θ is its parameter vector.

The predicted power ramp rate (slope) is approximated from the forecast trajectory as

(10) $$S_{p}\left(t\right)\approx \frac{\hat{P}\left(t+\Delta \right)-P\left(t\right)}{\Delta }$$

where $P\left(t\right)$ is the measured active power at time t. Large $\left|S_{p}\left(t\right)\right|$ indicates sharp power ramps and stronger electrical stress on the DFIG. Based on the unified electro-mechanical model and DC-link dynamics, two physical risk channels are defined:

Overcurrent risk channel. The predicted power slope $\left|S_{p}\left(t\right)\right|$ affects slip dynamics and the amplification of rotor current. Together with the current safety limit $I_{safe}$ (rated rotor-current limit), it is used to construct an overcurrent risk index.

Overvoltage risk channel. The predicted power imbalance between the rotor-side converter (RSC) and grid-side converter (GSC) determines the charging or discharging of the DC-link capacitor. Combined with the DC-link voltage safety limit $V_{safe}$, this yields an overvoltage risk index.

These two channels translate forecast information $\left(\hat{P}\left(t+\Delta \right),S_{p}\left(t\right)\right)$ into quantities directly related to hardware constraints (rotor current and DC-link voltage).

#### 3.3.2. Normalized Risks and Composite Risk Indicator

To make the different risk indices dimensionless and comparable, all channels are normalized to the interval [0, 1]. Let ${\hat{I}}_{peak}\left(t\right)$ denote the forecast rotor–current peak and $\;{\hat{V}}_{dc,peak}\left(t\right)$ the forecast DC–link voltage peak within the look-ahead window [t, t + Δ]. Using the hardware safety limits $I_{lim}$ and $V_{lim}$, the normalized overcurrent and overvoltage risks are defined as

(11) $$\left\{\begin{array}{c} r_{I}\left(t\right)=\min\left(\frac{{\hat{I}}_{peak}\left(t\right)}{I_{lim}},\;1\right)\\ r_{V}\left(t\right)=\min\left(\frac{{\hat{V}}_{dc,peak}\left(t\right)}{V_{lim}},\;1\right) \end{array}\right.\;$$

In addition, two auxiliary indices are introduced. Let $\hat{s}\left(t\right)$ be the forecast power ramp rate and $\sigma_{res}\left(t\right)$ a residual-based measure of forecast uncertainty. With reference values $s_{ref}$ and $U_{ref\;}$, they are normalized as

(12) $$\left\{\begin{array}{c} r_{S}\left(t\right)=\min\left(\frac{\left|\hat{s}\left(t\right)\right|}{s_{ref}},\;1\right)\\ r_{U}\left(t\right)=\min\left(\frac{\sigma_{res}\left(t\right)}{U_{ref\;}},\;1\right) \end{array}\right.\;$$

Thus, $r_{I}\left(t\right)$, $r_{V}\left(t\right)$, $r_{S}\left(t\right),\;andr_{U}\left(t\right)$ are all clipped to the range [0, 1]. Any additional indicators, if needed, are normalized in the same way and saturated to [0, 1] as well. The composite risk indicator R(t) is then defined as

(13) $$R\left(t\right)=\sigma r_{I}\left(t\right)+\beta r_{V}\left(t\right)+\gamma r_{S}\left(t\right)+\delta r_{U}\left(t\right)$$

where α, β, γ, δ ≥ 0 are weighting coefficients satisfying α + β + γ + δ = 1.

In this work, these coefficients are selected according to both physical priority and numerical performance. Specifically, the overcurrent and DC-link overvoltage risks represent the primary electrical constraints of the DFIG and therefore dominate the composite indicator (σ+β≈0.7−0.8) while the ramp-intensity and forecast-uncertainty indices serve as auxiliary early-warning channels (γ+δ≈0.2−0.3). The final coefficient values used in the simulations are obtained by parameter scanning within these ranges and selecting the combination that yields the best LVRT stability and recovery characteristics. The adopted values are summarized in Table 2 for reproducibility.

#### 3.3.3. Adaptive Thresholds and Timing Design

Based on the composite risk indicator R(t), adaptive current and voltage limits and an adaptive crowbar holding time are designed as

(14) $$\left\{\begin{array}{c} I_{lim}\left(t\right)=I_{0}-k_{1}R\left(t\right)\\ V_{lim}\left(t\right)=V_{0}-k_{2}R\left(t\right)\\ T_{hold}\left(t\right)=T_{0}+k_{3}R\left(t\right) \end{array}\;\right.$$

The corresponding weighting and adaptive gain parameters adopted in this work are listed in Table 2, where $I_{0}$ and $V_{0}$ are baseline current and voltage thresholds, $T_{0}$ is the minimum crowbar holding time, and $k_{1},k_{2},k_{3}>0$ are adaptive gain coefficients that determine how strongly the limits and holding time are adjusted as the composite risk R(t) varies. These gains are tuned using the unified DFIG model to ensure a practical trade-off between rotor-current suppression, DC-link voltage stabilization, and reactive power support. In particular, $k_{1}\;$ and $k_{2\;}$ control the sensitivity of the current and voltage limits to the risk indicator, while $k_{3}$ defines the additional holding-time margin required for safe converter re-synchronization under high-stress conditions. Their specific values for the 9 MW case study are provided in Table 3, and the same tuning procedure can be applied for different wind farms or operating conditions.

The crowbar insertion logic is modified as follows: If the instantaneous rotor current $i_{r}\left(t\right)\;$ or DC-link voltage $V_{dc}\left(t\right)\;$ exceeds the adaptive limits $I_{lim}\left(t\right)$ or $V_{lim}\left(t\right)$, or if R(t) exceeds a high threshold $R_{high}$ within an allowable early-action window before the nominal fault instant, then the crowbar is pre-emptively triggered, the RSC is blocked, and the rotor circuit is shorted through the crowbar resistance.

The crowbar release logic requires that $i_{r}\left(t\right)\;$ and $V_{dc}\left(t\right)\;$ have both fallen below their adaptive limits, $V_{dc}\left(t\right)\;$ has remained within a narrow recovery band around its nominal value for a specified dwell time, and the composite risk indicator satisfies $R(t)<R_{low}$ and the elapsed time exceeds $T_{hold}\left(t\right)$.

**Figure 8:**
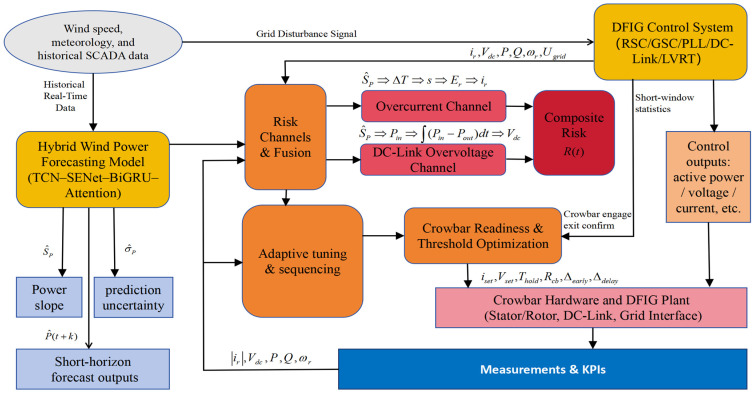
Framework of hybrid wind-power forecasting and forecast-driven crowbar coordinated control for DFIG-based wind turbines.

In this way, the forecast-driven strategy executes a sequence of “robust phase-lock → fast limiting → reinforced support → smooth recovery,” with crowbar actions coordinated by the predicted evolution of system stress rather than fixed thresholds. For clarity, the overall information flow from hybrid forecasting, through risk assessment, to adaptive crowbar coordination is summarized in Figure 8.

## 4. Simulation Setup and Experimental Design

### 4.1. DFIG Wind Turbine and Grid Model

To evaluate the proposed forecast-driven adaptive crowbar strategy, a 9 MW equivalent wind farm is modeled in MATLAB/Simulink [41]. The wind farm consists of six 1.5 MW DFIG wind turbines connected to a medium-voltage collection bus, a step-up transformer, transmission lines, and the upstream grid, forming a complete “turbine–DFIG–back-to-back converters–step-up transformer–grid” topology [42].

**Figure 9:**
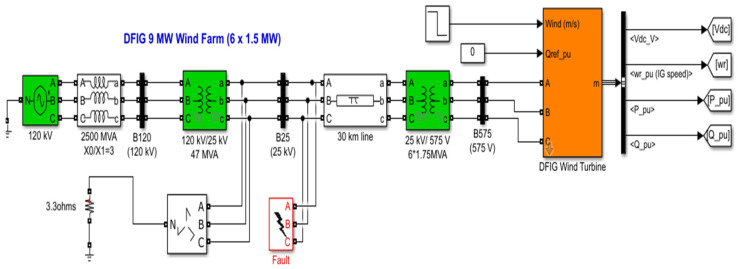
Simulation model of the 9 MW DFIG-based wind farm and grid connection.

Figure 9 shows the simulation model of the 9 MW DFIG-based wind farm and its grid connection. Each turbine is represented by an aero–mechanical drive-train model coupled to an electromagnetic DFIG in the αβ/dq frames. Rotor-side and grid-side converters adopt dual closed-loop control with outer power/voltage loops and inner current loops. Speed regulation and pitch control are implemented using standard structures commonly adopted in DFIG wind turbine studies.

**Figure 10:**
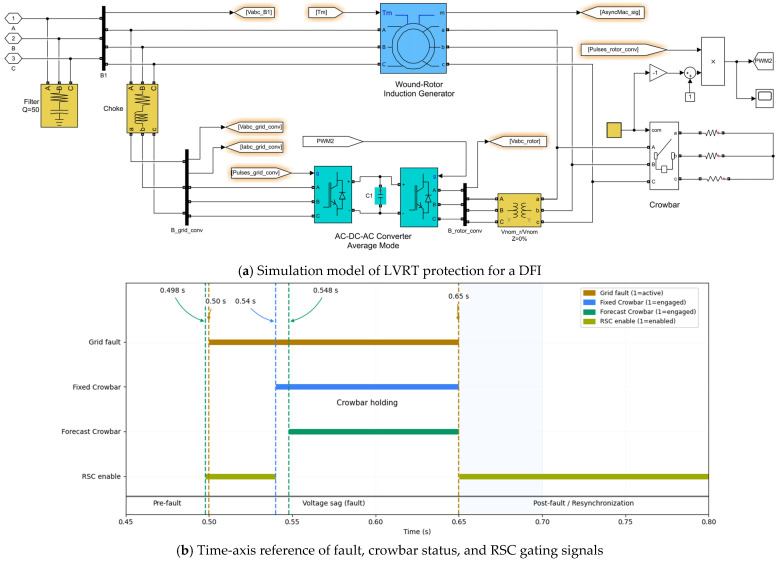
LVRT protection model and time-axis reference (**a**) and (**b**).

Low-voltage ride-through (LVRT) performance is studied by applying a three-phase short-circuit fault at the grid side. Unless otherwise specified, the fault starts at t = 0.50 s and is cleared at t = 0.65 s, producing a deep voltage sag at the point of common coupling. Figure 10a shows the LVRT protection model for the DFIG-based wind turbine, including crowbar insertion in the rotor circuit and coordinated converter control. Figure 10b gives the corresponding time-axis reference, indicating the pre-fault period, voltage sag, crowbar insertion/holding/release, and post-fault resynchronization.

The main electrical, mechanical, and fault-related parameters of the 9 MW model are listed in Table 3. The table focuses on quantities that have a direct impact on LVRT dynamics and crowbar design, such as rated power, base voltages, DC-link capacitance, crowbar resistance range, and fault depth and duration. Other control and filter parameters adopt conventional values used in DFIG LVRT studies.

### 4.2. Data Sources and Forecasting Task Definition

The forecasting experiments use SCADA data from a wind farm in Northwest China. The dataset covers approximately six months with a 15 min sampling interval and contains about 17,000 time steps. The main variables include active power, wind speed, wind direction, and several meteorological quantities such as temperature and air pressure. Before modeling, a quality-control procedure is applied, including removal of clearly erroneous records (e.g., negative power values during normal operation or obvious sensor failures) and interpolation-based repair of short missing segments.

Active power is taken as the forecast target. Wind speed, direction, and selected meteorological variables are used as auxiliary inputs. A sliding-window scheme is adopted: at each time t, a historical window of length L (corresponding to L × 15 min) is used as input to predict the power 1–4 steps ahead. In this study, the focus is on 15–60 min horizons, which are directly relevant to LVRT coordination and crowbar decision making.

For model training and evaluation, the six-month time series is split chronologically. The first 80% of the samples serve as the training set, the next 10% as the validation set for early stopping and hyperparameter selection, and the remaining 10% as the test set. Thus, the overall ratio between (training + validation) and test data is approximately 9:1. All models are trained and evaluated on exactly the same time partition, and no future information is allowed to enter the inputs of any test instance, ensuring a fair and reproducible comparison.

To couple the data-driven forecaster with the 9 MW DFIG simulation model, measured power trajectories from the SCADA dataset are used to shape the operating condition of the equivalent wind farm. A representative day with pronounced power variations is selected from the six-month record, and the corresponding active-power curve is scaled to the 9 MW rating. In the LVRT simulations, the three-phase fault is placed within a ramping interval of this scaled power trajectory. The hybrid forecaster provides 15–60 min ahead predictions of power and ramp rate around the fault instant, and these look-ahead quantities are used to construct the overcurrent and overvoltage risk indices and the composite risk indicator R(t), which determine the adaptive crowbar thresholds and timing in the LVRT studies.

### 4.3. Implementation Details and Evaluation Metrics

CEEMDAN decomposition and entropy analysis are implemented in MATLAB R2018b, while the deep-learning and XGBoost models are implemented in Python 3.9 using the PyTorch 2.2.2 and XGBoost 2.1.3. All experiments are run on a workstation equipped with an Intel i7 CPU; GPU acceleration is used for training the Informer and LSTM networks.

The following models are compared: ARIMA (Model 1), XGBoost (Model 2), LSTM (Model 3), LSTM+XGBoost (Model 4), Informer (Model 5), CEEMDAN–Informer (Model 6), CEEMDAN–Informer–LSTM–XGBoost (Model 7, proposed). Hyperparameters are tuned via grid search on the validation set, and input window length and forecast horizon are kept consistent across models.

Forecast performance is evaluated using four standard metrics: mean squared error (MSE), root mean squared error (RMSE), mean absolute error (MAE), and coefficient of determination R2. Their definitions follow conventional practice and are summarized in the caption of Table 4.

LVRT performance is evaluated using DC-link voltage peaks/valleys, rotor current peak, 95% active power recovery time, and reactive power peak, as summarized in Table 5.

## 5. Results and Analysis

### 5.1. Model Validation and Simulation Setup

In this study, the proposed hybrid forecast-enabled adaptive crowbar coordination strategy is primarily validated through simulations conducted using a 9 MW equivalent wind farm model in MATLAB/Simulink. The model incorporates a unified DFIG system with rotor- and grid-side converters, as well as the adaptive crowbar coordination strategy for LVRT enhancement under voltage sag conditions.

While simulation results are instrumental in validating the proposed approach, we acknowledge that real-world validation is essential for confirming its practicality and robustness. Therefore, future work will focus on validating the simulation model against real SCADA data from operational wind farms. This will allow us to assess the accuracy of the forecasted power, ramp rates, and the corresponding adaptive crowbar control strategies under actual operational conditions. Additionally, hardware-in-the-loop (HIL) testing will be performed to further validate the practical performance and integration of the adaptive crowbar coordination strategy with the DFIG wind turbines.

### 5.2. Hybrid Forecasting Performance on Wind Farm Data

Table 4 compares the forecasting performance of the seven models on the test set. The proposed hybrid model (Model 7) achieves the lowest MSE, RMSE, and MAE and the highest R^2^ among all models. Compared with the baseline ARIMA model, the MSE is reduced by roughly 90%; relative to XGBoost and LSTM, the reduction is around 88% and 85%, respectively. Even compared with the best single-branch deep model (Informer), the MSE reduction exceeds 70%.

Furthermore, compared with CEEMDAN–Informer (Model 6), the proposed model still yields a substantial improvement, indicating that adding an LSTM branch to enhance local temporal memory and introducing an XGBoost low-frequency channel for trend modeling both bring additional benefits beyond CEEMDAN + Informer alone.

Figure 8 shows the forecasting results of several representative models over a selected time window. The proposed model most accurately tracks the peaks, troughs, and ramps of the power series, with smaller phase lag and amplitude distortion. Figure 9 presents boxplots of absolute prediction errors for the different models. The proposed model exhibits the narrowest error distribution and the fewest outliers, which reflects both high accuracy and high robustness.

These results demonstrate that CEEMDAN effectively reduces signal complexity, and the dual-channel modeling strategy captures multi-scale patterns efficiently, providing forecasts of sufficient quality for real-time LVRT coordination.

### 5.3. LVRT Response and Crowbar Coordination Under Deep Voltage Sags

We now analyze the LVRT behavior of the 9 MW equivalent wind farm under a deep three-phase voltage sag and compare three strategies:

(1) No crowbar (No CB): converter control only, without hardware protection.
(2) Fixed-threshold crowbar (Fixed CB): conventional crowbar with fixed current/voltage thresholds and holding time.
(3) Forecast-driven crowbar (Proposed): an adaptive strategy that uses the composite risk indicator R(t) to adjust current and voltage limits and crowbar holding time in real time.

The time-axis reference for fault occurrence, crowbar insertion, and RSC gating is defined such that, under the proposed strategy, the crowbar is slightly pre-triggered relative to the nominal fault instant when the risk indicator rises, and its release is slightly delayed compared with the fixed-threshold strategy, with the early/late adjustments bounded by design and determined by the evolution of the risk indicator.

**Figure 11:**
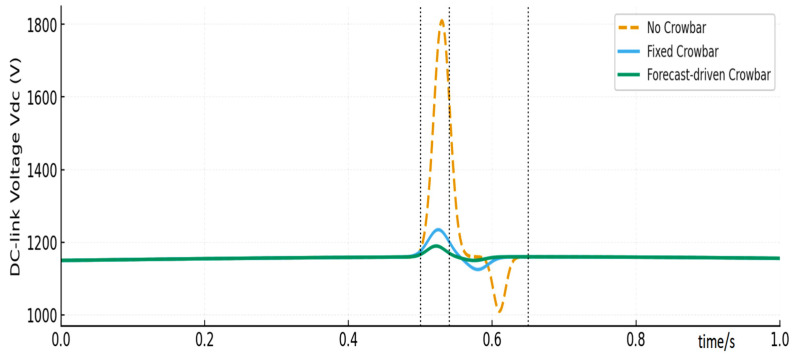
DC-link voltage comparison.

Figure 11 compares the DC-link voltage responses under the three strategies. In the No CB case, severe overvoltage spikes and oscillations occur, which are likely to trigger converter protections. In the Fixed CB case, the peak is mitigated but noticeable overshoot and undershoot remain after crowbar release. In contrast, the Proposed strategy keeps the DC-link voltage within approximately ±3% of its nominal value, with a smoother and more symmetric waveform. The DC voltage peaks and minima for each strategy are summarized in Table 4, along with relative improvements.

**Figure 12:**
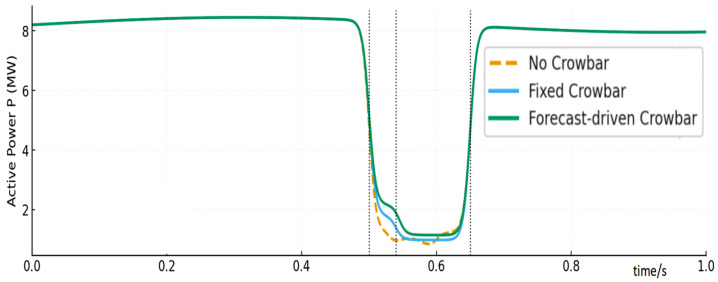
Active power comparison.

Figure 12 shows the active power trajectories. The Proposed strategy yields the fastest recovery: the 95% active power recovery time is reduced from about 0.35 s (No CB) and 0.22 s (Fixed CB) to about 0.18 s (Proposed), as listed in Table 4. This indicates better coordination between crowbar release and RSC re-synchronization.

**Figure 13:**
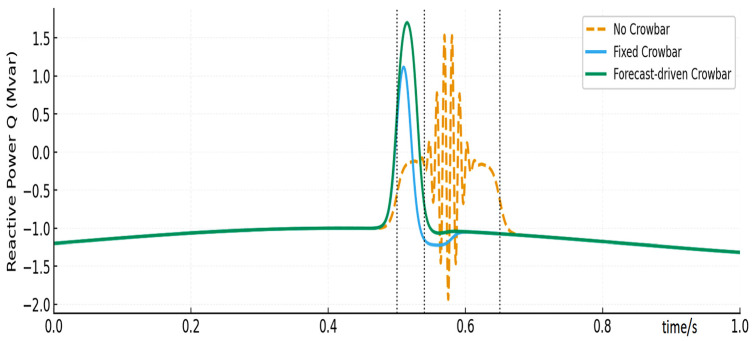
Reactive power comparison.

Figure 13 presents the reactive power responses during the fault. In the No CB case, reactive power oscillates strongly and is even negative in certain intervals, providing poor voltage support. The Fixed CB strategy supplies some positive reactive power but with noticeable fluctuations. The Proposed strategy achieves the highest and most stable reactive power peak (about 1.7 Mvar, consistent with the abstract), significantly reinforcing grid voltage support and complying well with LVRT requirements.

**Figure 14:**
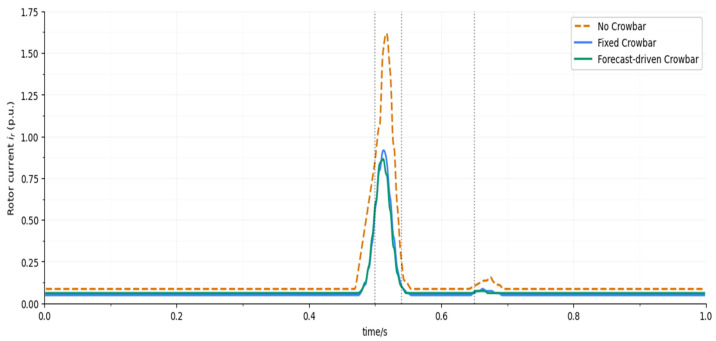
Rotor current comparison.

Figure 14 compares the rotor current waveforms. In the No CB case, the peak reaches around 1.75 p.u., posing a serious threat to converter safety. The Fixed CB strategy reduces the peak to about 0.98 p.u., but a small “bump” remains at crowbar release. The Proposed strategy further reduces the peak to about 0.93 p.u., with a smoother decay, thanks to forecast-driven early limiting and adaptive holding time. The quantitative values and relative improvements are listed in Table 4.

Overall, the forecast-driven crowbar strategy significantly enhances LVRT performance across multiple dimensions: it improves DC-link voltage stability, suppresses rotor current peaks, accelerates active power recovery, and strengthens reactive support, all without any hardware modification.

### 5.4. Sensitivity and Ablation Analyses

To better understand the contribution of each component to the overall performance, sensitivity and ablation studies are conducted.

First, regarding the forecasting module, Table 3 and Figure 8 and Figure 9 provide a clear implicit ablation analysis. ARIMA and XGBoost serve as traditional statistical and machine learning baselines, while LSTM, LSTM+XGBoost, and Informer represent single-branch deep models. CEEMDAN–Informer (Model 6) is a decomposition-based, single-branch hybrid model. By comparing Models 1–6 with Model 7, the following observations are made:

CEEMDAN Decomposition (Model 6 vs. Model 5): The decomposition provided by CEEMDAN substantially reduces both MSE and MAE. This improvement is achieved by effectively separating high-frequency fluctuations (noise) from low-frequency trends, allowing the model to capture the underlying patterns more accurately.

Adding the LSTM Branch (Model 7 vs. Model 6): Incorporating the LSTM branch further enhances local temporal modeling, particularly during fast ramps. This improvement becomes evident in the zoomed-in view shown in Figure 8 (inset), where the model’s superior performance during rapid changes in wind power is clearly visible. The LSTM component helps track short-term dynamics, reducing phase lag and improving response time.

Introducing the LF XGBoost Channel (Model 7 vs. Model 6): The addition of the XGBoost channel for low-frequency components further corrects long-term trends and residuals, resulting in the best overall performance. This hybrid approach significantly improves the model’s ability to capture both short-term and long-term dynamics, yielding more reliable forecasts.

To provide a more detailed comparison, Figure 8 presents a zoomed-in view of the critical time window, highlighting the differences in performance between the models during rapid wind power fluctuations. The inset clearly demonstrates how the combination of CEEMDAN decomposition, LSTM, and XGBoost channels leads to improved forecasting accuracy during fast wind power ramps.

Second, for protection parameters, we vary the crowbar resistance $R_{cb}$ within and beyond the feasible range suggested by the unified model and scan the risk weights α,β,γ, and δ in the composite risk indicator R(t). The results indicate that:

If $R_{cb}$ is too small, rotor current suppression remains insufficient and DC-link overvoltage is aggravated even under adaptive thresholds;

If $R_{cb}$ is too large, rotor current peaks are low but DC voltage recovers slowly and reactive support deteriorates;

When $R_{cb}$ takes moderate values in the 0.4–0.8 p.u. range and is combined with appropriate adaptive gains $k_{1}$, $k_{2}$, $k_{3}$, an optimal trade-off among current limiting, voltage stabilization, and reactive support is achieved.

Third, we evaluate robustness to forecasting errors and uncertainty by perturbing the forecast outputs and observing changes in the composite risk R(t) and crowbar actions. Thanks to the normalization and smoothing used in the definition of R(t) occasional forecast spikes do not trigger spurious actions; only when power ramps and energy imbalances persist over the look-ahead window does the risk accumulate sufficiently to prompt adaptation. This suggests that the designed risk channels and smoothing strategy provide a robust bridge between probabilistic forecasting and hardware protection.

## 6. Discussion

This work explores the integration of short-term wind power forecasting with DFIG LVRT protection through a forecast–protection–control loop. Compared to conventional crowbar schemes that rely on fixed current and voltage thresholds, the proposed strategy interprets forecasted power, ramp rates, and residual-based uncertainty as overcurrent and DC-link overvoltage risks. These risks are combined into a composite indicator, which is used to adjust crowbar limits and holding time in real-time. The case study suggests that using look-ahead information in this way helps align protection actions with the evolving stress on the turbine–converter system, rather than reacting solely to instantaneous measurements.

From the forecasting perspective, the CEEMDAN–Informer–LSTM–XGBoost model aligns with the general trend of decomposition-based deep learning approaches, but introduces two additional elements. First, sample entropy is used to characterize the complexity of each intrinsic mode function (IMF), distinguishing high-entropy fluctuations from low-entropy trends. Second, a dual-stream structure is adopted, combining long-range attention with local temporal memory, while a tree-based model is applied to low-frequency behavior. Comparative results against statistical baselines (ARIMA, XGBoost), single-branch deep models (LSTM, Informer), and a CEEMDAN–Informer variant demonstrate that allocating model capacity based on component complexity is a reasonable design choice, though it does increase the complexity of the training pipeline.

In the 9 MW equivalent wind-farm simulations, the forecast-driven crowbar coordination yields more stable DC-link voltage trajectories, lower rotor-current peaks, faster active-power recovery, and stronger reactive support during a representative deep voltage sag than the two benchmark strategies. Parameter scans on crowbar resistance and risk weights suggest that a moderate resistance range, coupled with suitable adaptive gains, provides a balanced compromise between current limiting, voltage behavior, and reactive support capability. Robustness checks, in which forecast outputs are perturbed, show that the normalized and smoothed risk indicator is relatively insensitive to isolated forecast spikes, responding primarily to sustained ramps and energy imbalances, which is desirable for practical protection logic.

Several limitations must be acknowledged. The forecasting module provides mainly point estimates, and uncertainty is only indirectly reflected through residual statistics. More explicit probabilistic modeling could enable clearer risk quantification. The LVRT assessment is limited to a 9 MW equivalent wind farm under a restricted set of deep three-phase fault scenarios, without systematic coverage of asymmetric faults, repeated disturbances, or very weak grids. Additionally, the coordination strategy is evaluated at the single-farm level, without considering interactions among multiple wind plants or with auxiliary devices such as STATCOMs and storage. Future research could incorporate quantile or interval forecasts into the risk channels, validate the strategy using hardware-in-the-loop platforms and field data under diverse fault types, and extend the forecast–risk–control framework to cluster-level coordination and multi-device interactions.

In comparison with previous studies, such as Hu et al. (2022) [38], which utilized a CEEMDAN-LSTM-TCN model for ultra-short-term wind power forecasting, our approach integrates forecasted information into an adaptive control loop for DFIG systems, improving real-time LVRT protection. Similarly, Sabzevari et al. (2023) [17] focused on enhancing LVRT capability under symmetrical and asymmetrical faults in DFIG systems using FO-PID and RCO techniques. Our method extends this by incorporating forecasting-driven coordination, enabling more adaptive and efficient protection, improving LVRT performance, and providing real-time adjustments based on predictive results.

## 7. Conclusions

This paper presents a hybrid forecast-enabled adaptive crowbar coordination strategy for DFIG wind turbines, integrating short-term wind power forecasting with LVRT protection and converter control. The key conclusions are as follows:

(1) Forecast–protection–control loop for DFIG LVRT: A novel forecast–protection–control loop for DFIG LVRT is proposed. A unified DFIG–converter–crowbar model and LVRT framework are developed for a 9 MW equivalent wind farm. Forecasted power, ramp rate, and residual-based uncertainty are mapped into overcurrent and DC-link overvoltage risk indices, which are then combined into a time-varying composite risk indicator. This indicator is used to adjust crowbar current and voltage limits, as well as holding time, ensuring that crowbar insertion and release are synchronized with the anticipated system stress.
(2) Entropy-aware forecasting model: An entropy-aware CEEMDAN–Informer–LSTM–XGBoost model improves short-term wind power prediction quality. CEEMDAN is employed to decompose the time series into multi-scale components, and sample entropy is used to characterize their complexity. High-entropy, high-frequency components are modeled using a dual-stream Informer–LSTM structure, while low-entropy, low-frequency components are handled with an XGBoost trend channel. The hybrid model, evaluated on six months of wind farm data, outperforms several statistical, machine learning, and deep learning baselines in terms of error metrics and goodness-of-fit, providing forecasts that are suitable for real-time LVRT coordination.
(3) Improved LVRT responses with forecast-driven crowbar strategy: The forecast-driven crowbar strategy enhances LVRT performance in the simulated case study. During a deep three-phase voltage sag in the 9 MW equivalent wind farm, the proposed strategy results in more controlled DC-link voltage behavior, reduced rotor-current peaks, faster active power recovery, and enhanced reactive power support compared to both no-crowbar protection and fixed-threshold crowbar schemes. These improvements demonstrate that integrating short-term predictive information into crowbar coordination is a promising direction for enhancing LVRT performance in DFIG-based wind power systems.
(4) Promising approach for system-level improvement: These results suggest that combining decomposition-based hybrid forecasting with risk-oriented protection design offers a promising approach to improving the dynamic behavior of DFIG wind turbines under grid faults. This strategy motivates further exploration under broader operating conditions and more diverse system configurations, including multi-turbine coordination and interactions with auxiliary devices.

## Figures and Tables

**Table 1:** Sample entropy of CEEMDAN derived IMFs and complexity ranking.

Rank	IMF	Sample Entropy (SampEn)
1	IMF1	0.595240
2	IMF3	0.544799
3	IMF2	0.533649
4	IMF4	0.523960
5	IMF5	0.415241
6	IMF6	0.251982
7	IMF7	0.107578
8	IMF8	0.044777
9	IMF9	0.019227
10	IMF10	0.008408
11	IMF11	0.001856

**Table 2:** Selected weighting and adaptive gain parameters for the proposed crowbar coordination strategy.

Parameter	Meaning	Selected Value	Source
α	Weight of rotor overcurrent risk	0.40	Numerical scan
β	Weight of DC-link overvoltage risk	0.35	Numerical scan
γ	Weight of ramp-intensity index	0.15	Numerical scan
δ	Weight of forecast uncertainty index	0.10	Numerical scan
$k_{1}$	Adaptive gain for current limit	0.25	Unified model + scan
$k_{2}$	Adaptive gain for voltage limit	0.15	Unified model + scan
$k_{3}$	Adaptive gain for holding time	80 ms	Unified model + scan

**Table 3:** Main parameters of the 9 MW DFIG wind farm and LVRT fault scenario.

Category	Parameter	Symbol	Value/Setting	Unit/Note
Wind farm configuration	Number of WTGs	N	6	–
	Rated power per WTG	$P_{n}$	1.5	MW
	Total rated power	$P_{farm}$	9	MW
Rated electrical values	Stator nominal line voltage	$V_{s,nom}$	575	$V_{rms}$
	Rotor nominal line voltage	$V_{r,nom}$	1975	$V_{rms}$
	Grid frequency	$f_{s}$	60	Hz
DFIG stator parameters	Stator resistance	$R_{s}$	0.023	p.u
	Stator inductance	$L_{s}$	0.18	p.u
DFIG rotor/magnetizing	Rotor resistance	$R_{r}$	0.016	p.u
	Rotor leakage inductance	$L_{r}$	0.16	p.u
	Magnetizing inductance	$L_{m}$	2.9	p.u
Mechanical parameters	Inertia constant	H	0.685	s
	Friction factor	F	0.01	–
	Number of pole pairs	P	3	–
	Initial slip	$s_{0}$	−0.2	–
DC-link & converter	DC-link capacitance	$C_{dc}$	0.01	F
	Nominal DC-link voltage	$V_{dc,ref}$	1150	V
Fault scenario	Fault type	–	Three-phase short circuit at grid side	Deep voltage sag
	Fault start time	$t_{f}$	0.50	s
	Fault clearing time	$t_{c}$	0.65	s
Crowbar & LVRT settings	Crowbar resistance range	$R_{cb}$	0.4–0.8	p.u
	Nominal crowbar resistance (case study)	$R_{cb}^{*}$	0.6	p.u
	Minimum holding time	$T_{0}$	20	ms
	Action-decision time window	$T_{arm}$	5–10	ms
	Adaptive gain for current limit	$k_{1}$	0.25	–
	Adaptive gain for voltage limit	$k_{2}$	0.15	–
	Delay gain/late-release factor	$k_{3}$	80	ms

**Table 4:** Prediction Performance Comparison of Model Variants.

DATE	Model	MSE	RMSE	MAE	R^2^
October 2019–March 2020	ARIMA(1#)	6.6343	2.5757	2.0732	0.7934
	XGBoost(2#)	5.4925	2.3436	2.0041	0.8290
	LSTM(3#)	4.3903	2.0953	1.6732	0.8633
	LSTM+XGBoost(4#)	3.7908	1.9470	1.6511	0.8820
	Informer(5#)	2.7984	1.6728	1.3812	0.9129
	CEEMDAN–Informer(6#)	1.9681	1.4029	1.1482	0.9387
	CEEMDAN–Informer–LSTM+XGBoost(7#)	0.6384	0.7990	0.6033	0.9801

#: Model index corresponding to different prediction methods used in this study.

**Table 5:** Key LVRT metrics and improvement rates for the forecast-driven crowbar.

Metric	No Crowbar	Fixed Crowbar	Forecast-Driven CB	Improvement vs. Fixed (%)	Improvement vs. No-CB (%)
$V_{dc\_peak}$ (050–0.65 s)	1810	1210	1188	↓ 1.8%	↓ 34.4%
$V_{dc\_min}$ (050–0.65 s)	1010	1130	1145	–	–
${peak\;i}_{r}$ (p.u.)	1.75	0.98	0.93	↓ 5.1%	↓ 46.9%
P recovery to 95% (ms)	40	22	18	↓ 18.2%	↓ 55.0%
$Q_{peak,capacitive}$ (Mvar)	1.5	1.1	1.7	↑ 54.5%	↑ 13.3%

Note: ↓ and ↑ denote a decrease and an increase in the metric compared with the reference case, respectively.

## Data Availability

The data used to support the findings of this study are available from the corresponding author upon request.

## Abbreviations

Abbreviation	Full Form
AGC	Automatic Generation Control
AVC	Automatic Voltage Control
BiGRU	Bi-directional Gated Recurrent Unit
BiLSTM	Bi-directional Long Short-Term Memory
CEEMDAN	Complete Ensemble Empirical Mode Decomposition with Adaptive Noise
CNN	Convolutional Neural Network
CP	Power Coefficient
DFIG	Doubly Fed Induction Generator
DL	Deep Learning
FACTS	Flexible AC Transmission System
GRU	Gated Recurrent Unit
GSC	Grid-Side Converter
GWEC	Global Wind Energy Council
LSTM	Long Short-Term Memory (Model)
LVRT	Low Voltage Ride Through
MAE	Mean Absolute Error
ML	Machine Learning
MPPT	Maximum Power Point Tracking
MSE	Mean Squared Error
PCA	Principal Component Analysis
PI	Proportional Integral
PLL	Phase Locked Loop
RL	Reinforcement Learning
RMSE	Root Mean Squared Error
RSC	Rotor-Side Converter
SCADA	Supervisory Control and Data Acquisition
STATCOM	Static Synchronous Compensator
SVPWM	Space Vector Pulse Width Modulation
TCN	Temporal Convolutional Network
VMD	Variational Mode Decomposition
VSCF	Variable Speed Constant Frequency
WECS	Wind Energy Conversion System
WPF	Wind Power Forecasting
WT	Wind Turbine
p.u.	per unit
